# Connecting scientists and oncologists for advances

**DOI:** 10.1016/j.cpt.2024.06.006

**Published:** 2024-07-01

**Authors:** Yunlong Yang, Changhao Wu, Wei Zhu

**Affiliations:** Department of Cellular and Genetic Medicine, School of Basic Medical Sciences, Fudan University, Shanghai 200032, China; Department of Biochemistry and Physiology, Faculty of Health and Medical Sciences, University of Surrey, Guildford GU2 7HX, UK; Department of Oncology, Jiangsu Province Hospital, And Nanjing Medical University First Affiliated Hospital, Nanjing, Jiangsu 210029, China

**Keywords:** Translational research, Cancer pathogenesis, Cancer diagnosis, Cancer therapy, Adverse effects

This special issue, titled **“**Basic and translational research on oncology**”**, in the founding year of *Cancer Pathogenesis and Therapy* aims to represent the broad scope of this ambitious young journal. With a deeper understanding of cancer biology, oncology has advanced at an impressive pace over the last decade, accompanied by continuous progress in cancer therapy. How do we, as scientists and oncologists, build on these foundations and milestones in such a rapidly changing academic frontier? How can we translate our understanding of molecular mechanisms to further contribute to cancer prevention, diagnosis, and personalized treatment? To address these aspects, in this issue, we present a series of recent advances in cancer pathogenesis, diagnosis, and treatment, and explore new methods that closely integrate basic research with clinical practice.

Cancer pathogenesis involves genomic, epigenetic, and proteomic changes in cancer cells, along with their pathological consequences. During cancer pathogenesis, the dysfunction of RNA splicing machinery profoundly alters the transcriptomic landscape. As a dynamic entity, the RNA splicing process requires spatiotemporal regulation that underlies its regulatory mechanisms and cancer-related consequences. Toward this noteworthy step, Yan et al.^1^ summarized the RNA splicing dysregulation landscape in lung cancer, positioning targeted RNA splicing as a therapeutic option.

With a deeper understanding of cancer molecular pathology and the relentless upgrades in nucleic acid sequencing technology, the molecular diagnostic revolution has transformed clinical oncology. In the field of molecular diagnostics, Li et al.^2^ established a diagnostic model based on newly identified negatively correlated microRNA (miRNA)/messenger RNA (mRNA) pairs that could be used to distinguish breast cancer from benign breast disease. Yu et al.^3^ conducted an epidemiological study to investigate *breast cancer gene 1/2* (*BRCA1/2*) mutation penetrance in a large cohort of the SIGHT study in East China, providing guidance for breast cancer prevention in the Chinese population. Beyond the improvements in molecular diagnostic equipment, advances in diagnostic software rely on new algorithms when facing immense amounts of cancer genomic data. Lu^4^ discussed somatic copy number alteration (CNA) detection for tumor evolution tracing and structuring of cancer phylogenetics, comparing various existing models that could potentially guide early cancer detection, prevention, and treatment.

Cancer treatment is a complex and challenging endeavor. Among the various cancer treatment alternatives, targeted therapy demonstrated breakthrough potential, offering robust and selective cancer inhibition with less damage to healthy tissues. Shukla et al.^5^ commented on the promising results of dostarlimab, a novel immunotherapeutic drug for endometrial cancer treatment. Jin et al.^6^ conducted a clinical trial to explore the efficacy and safety of a combination regimen with the new anti-human epidermal growth factor receptor 2 (HER2) drug inetetamab, yielding encouraging results. Other cancer therapeutic approaches are also of interest to this journal. For certain types of bladder cancer, intravesical installation with Bacillus Calmette-Guérin (BCG) is a valuable therapeutic strategy. Maroof et al.^7^ summarized the progress concerning this approach and discussed new strategies to rectify BCG treatment failure. Zhang et al.^8^ explored bone metastasis patterns following the surgical treatment of gastric cancer and its relation to patient survival in a retrospective analysis to inform more rational clinical management. In addition to treating the cancer itself, understanding and minimizing the adverse effects of cancer therapy is an integral part of cancer management. Wu and Chen^9^ discussed how a ketogenic diet could impact chemotherapy-induced thrombocytopenia. Rachma et al.^10^ summarized the pathogenesis and management plan of chemotherapy-induced cardiotoxicity. Developing precise and effective therapies will markedly contribute to oncology research and benefit cancer patients.

Importantly, most special issues only pursue interesting points of view rather than leading them. A plethora of emerging technologies and exciting discoveries should be brought to the broad readership of this journal via subsequent editorial work. We at *Cancer Pathogenesis and Therapy* thank the authors and reviewers for their contribution and hope that all the readers will enjoy reading this special issue.

## Authors contribution

Yunlong Yang, Changhao Wu, and Wei Zhu wrote the manuscript and were responsible for the critical review of the manuscript. All the authors have read and approved the final version of the manuscript.

## Ethics statement

None.

## Declaration of generative AI and AI-assisted technologies in the writing process

The authors declare that generative artificial intelligence (AI) and AI assisted technologies were not used in the writing process or any other process during the preparation of this manuscript.

## Funding

None.

## Data availability statement

All the data are included in the manuscript.

## Conflict of interest

The authors declare that they have no known competing financial interests or personal relationships that could have appeared to influence the work reported in this paper.
